# Systematic review of the incidence of post-operative trichiasis in Africa

**DOI:** 10.1186/s12886-020-01564-0

**Published:** 2020-11-17

**Authors:** Grace Mwangi, Paul Courtright, Anthony W Solomon

**Affiliations:** 1 Department of Surgery, Division of Ophthalmology, University of Cape Town, Cape Town, South Africa; 2 Kilimanjaro Centre for Community Ophthalmology, Division of Ophthalmology, University of Cape Town, Cape Town, South Africa; 3 Department of Control of Neglected Tropical Diseases, World Health Organization, Geneva, Switzerland

**Keywords:** Trachoma, Trichiasis, Entropion, Post-operative trichiasis, Incidence, Eyelid diseases, Surgery

## Abstract

**Background:**

Surgery for trichiasis is one of the pillars of the World Health Organization’s strategy for global elimination of trachoma as a public health problem. A high incidence of post-operative trichiasis or other poor surgical outcomes could jeopardize these efforts. In this review, we aimed to summarize the reported incidence of post-operative trichiasis and other poor outcomes of trichiasis surgery in Africa.

**Methods:**

We conducted a systematic literature search using PubMed, Academic Search Premier, Africa-Wide Information, CINAHL and Health Source Nursing through EBSCOhost, Web of Science, and the Cochrane Central Register of Controlled Trials. Reference lists of included studies were also reviewed to identify further potentially relevant publications. All observational and interventional studies that measured post-operative trichiasis in Africa as an outcome of trichiasis surgery were included.

**Results:**

Thirty-five papers reporting on 22 studies (9 interventional,13 observational; total 13,737 participants) met the inclusion criteria. The reported incidence of post-operative trichiasis in the included studies ranged from 2% (at 6 weeks after bilamellar tarsal rotation) to 69% (at 3 weeks after anterior lamellar repositioning). The incidence varied by surgical procedure, study design, and length of follow-up.

**Conclusion:**

Trichiasis surgical outcomes should be improved. National trachoma programmes could benefit from identifying and adopting strategies to improve the performance and quality of their surgical service.

## Background

Trachoma is the leading infectious cause of blindness worldwide. It is caused by particular strains of the intracellular bacterium *Chlamydia trachomatis*, believed to be transmitted through infected eye and nose secretions carried on fingers, fomites and eye-seeking flies [1, 2]. Repeated infection leads to scarring of the tarsal conjunctiva, which can then lead to trichiasis (eyelashes touching the eyeball) [3]. Left untreated, trachomatous trichiasis can result in the formation of corneal opacities, which irreversibly impair vision [4].

Surgery is an integral part of the SAFE strategy (Surgery, Antibiotics, Facial cleanliness, Environmental improvement) advocated by World Health Organization (WHO) for elimination of trachoma as a public health problem [5]. Surgery for trichiasis aims to re-position the eyelid margin by externally rotating it so that eyelashes no longer touch the eyeball. Not all cases of trichiasis are due to trachoma and not all cases of trachomatous trichiasis involve entropion; other management approaches may be preferable when correction of entropion is not indicated. When appropriately performed, apart from preserving vision, surgery has also been shown to enhance patient comfort due to decreases in pain and photophobia, and resolution of corneal swelling [6]. Surgery substantially increases quality of life for individuals with trichiasis, even if vision itself does not improve [7]. However, for surgery to be effective in preventing trachoma-related blindness and to encourage other trichiasis patients to consent to an operation, there must be good long-term surgical outcomes with a low incidence of post-operative trichiasis.

WHO’s third global scientific meeting on trachoma [8], convened in 2010, recommended that national trachoma programmes should report the incidence of post-operative trichiasis, and target a cumulative post-operative trichiasis incidence of ≤10% at one-year post-surgery. However, several studies report incidence estimates that are considerably higher than this [9]. There are only limited operational data on the incidence of post-operative trichiasis published from settings where trachoma elimination programmes are active [9].

Generating an evidence-based understanding of the magnitude and determinants of post-operative trichiasis would help inform discussions on current outcome targets and strategies to improve surgery outcomes for trichiasis. This review aims to partially fill this gap by consolidating data on incidence of post-operative trichiasis and other poor outcomes of trichiasis surgery from observational and interventional studies conducted in Africa.

## Methods

### Ethical approval

The protocol was approved by the University of Cape Town Faculty of Health Sciences’ Human Research Ethics Committee (076/2018) and deemed by the WHO Ethics Review Committee to not require full formal ethics review (0003034). The protocol was registered on PROSPERO: CRD42018085253.

### Search strategy

We undertook a systematic search of PubMed, Academic Search Premier, Africa-Wide Information, CINAHL and Health Source Nursing through EBSCOhost, Web of Science [all databases], and the Cochrane Central Register of Controlled Trials, including material published up to and including the month of May 2018. In addition, Google Scholar and the reference lists of relevant reviews and all eligible papers were searched to cross-check for studies not already identified. Websites of organizations identified in published studies were searched for pertinent grey literature. No language limitations were applied in these searches.

The full search strategy is provided as a supplemental file.

### Study selection

Two reviewers (GM, PC) independently screened the titles and abstracts of the articles found to determine their potential eligibility for inclusion. The full texts of potentially eligible studies were obtained. Selection for inclusion into the review was conducted by the two reviewers working independently. Any disagreements regarding inclusion of studies were resolved by discussion or by consulting the third reviewer (AWS).

Included studies met the following criteria: 1) conducted within Africa; 2) measured post-operative trichiasis (defined as the presence of one or more eyelashes touching the eyeball or evidence of epilation of in-turned eyelashes after surgery) as an outcome; 3) included participants aged ≥15 years with previously unoperated trachomatous trichiasis in at least one eye within either [a] an interventional or [b] observational study.

Editorial articles, reviews, expert opinion pieces, conference papers and meeting abstracts were excluded, as were studies conducted outside Africa, studies that did not have a surgical intervention, and those that did not measure or report post-operative trichiasis as an outcome. Any studies published before 1990 were also excluded.

We divided studies into two groups - observational and interventional studies - with the expectation that surgeons operating in the latter were more likely to be highly selected and re-trained before the study than those in the former. It should be noted however, that some interventional studies included in this review did not report criteria-based selection or re-training of surgeons before the intervention.

### Qualitative assessment of studies

The methodological quality of the papers included in this review was assessed using a slight adaptation of the Joanna Briggs Institute critical appraisal checklist for prevalence studies [10]. Critical appraisal was conducted by two reviewers independently, with disagreements resolved through discussion or by consulting the third reviewer.

### Assessment of heterogeneity

Studies included in this systematic review were checked for heterogeneity by examining their characteristics (study design, population, follow up period, etc.) and risk of bias. Statistical heterogeneity was assessed using I^2^ and Chi [2] tests. Heterogeneity was considered as significant if the I^2^ was above 50%.

### Data synthesis

The studies included in this review were varied in terms of design, interventions, follow up period and outcomes. As a result, there was high statistical heterogeneity which precluded pooling of data for the outcomes (I^2^ > =95%). RevMan 5.3 software [11] was used for all quantitative data analyses in this review.

For the intervention studies, treatment effects were measured by calculating odds ratios for the different interventions and 95% confidence interval for these. Dichotomous data from these studies are presented in forest plots without a summary estimate, and as a narrative summary. For observational studies, data are presented as a percentage of those who developed the outcome of interest out of the total number of study participants.

## Results

### Results of the search

**Fig. 1:**
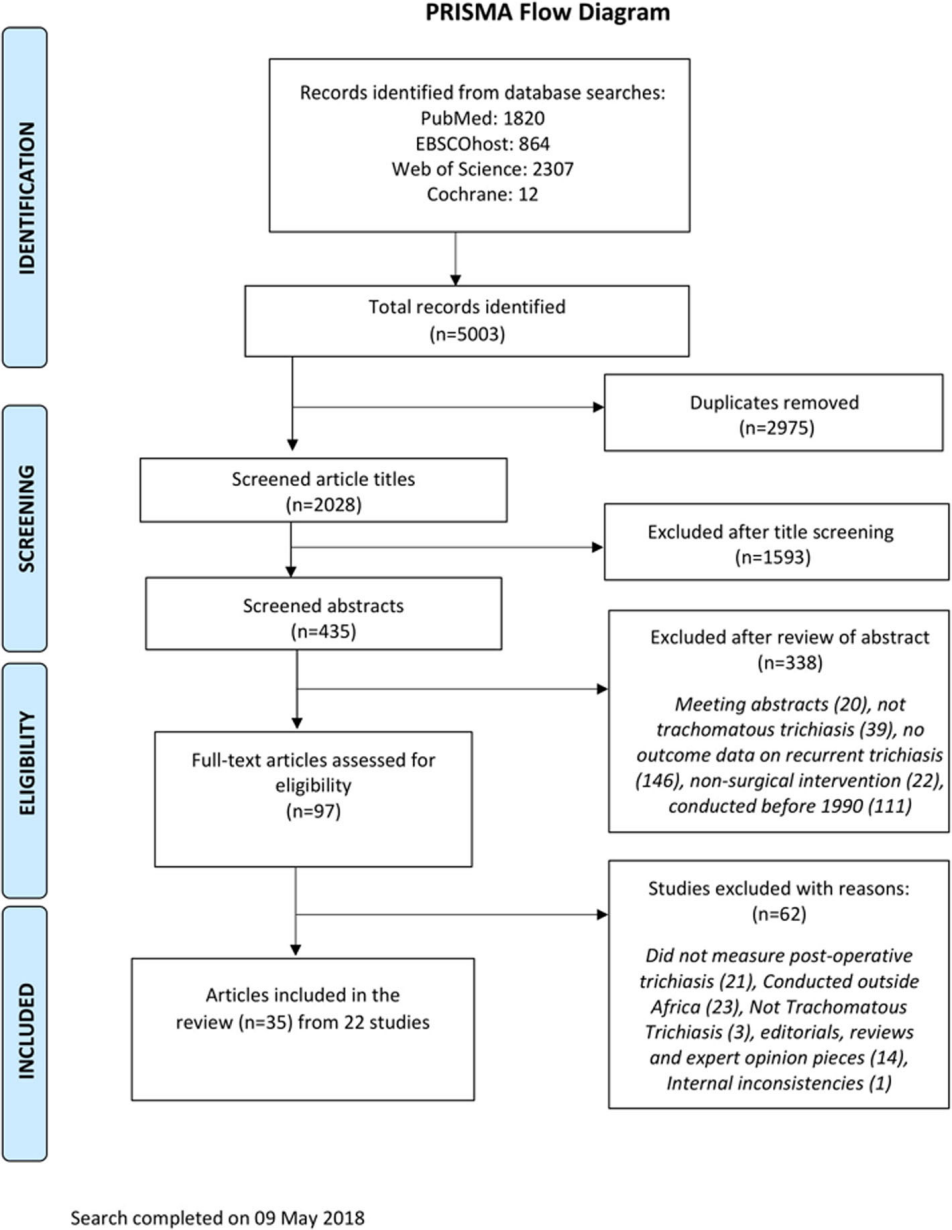
PRISMA Flow diagram

Initial electronic literature searches yielded 5003 articles. After removal of duplicates and a review of titles and abstracts of these and other articles identified by hand-searching, 97 publications were selected for detailed review and possible inclusion. Of these, 61 articles were excluded for the following reasons: conducted outside Africa (*n =* 23); did not measure post-operative trichiasis as an outcome (*n* = 21); the condition of interest was not trichiasis due to trachoma (*n* = 3); the article type was a review, editorial or expert opinion piece (*n* = 14). One prospective study [12] was subsequently also excluded from the review, despite having met the inclusion criteria, due to internal inconsistencies in the description of study methods and findings. Ultimately, 35 articles describing 22 studies (nine interventional and 13 observational studies with a combined total of 13,737 participants) were included. Figure 1 summarizes the flow of the paper identification and selection process. A list of excluded studies is provided as a Supplementary file.

### Study characteristics and quality

Table 1 summarises the characteristics and quality of the 22 studies that met the inclusion criteria. In the 22 studies, the duration of follow-up ranged from 3 weeks to more than 7 years. The incidence of post-operative trichiasis varied widely, from 2.3% [18] to 65% [33] (Table 1). Bilamellar tarsal rotation (BLTR) and posterior lamellar tarsal rotation (PLTR; sometimes referred to as the Trabut procedure) were the most commonly reported procedures, used in 16 and 13 studies respectively. Other procedures used were anterior lamellar rotation; transverse tarsotomy and lid margin rotation; and lid margin split [13, 29, 32]. We included all eligible studies regardless of the surgical procedure used, though BLTR and PLTR are the two procedures recommended by WHO for treatment of trachomatous trichiasis [47]. Twenty of the 22 studies included in this review had relatively low risk of bias from the design, conduct and analysis standpoint. Two observational studies [32, 38] had relatively small sample sizes which might have affected their study power. Findings from those two studies should therefore be interpreted with caution.

**Table 1:** Characteristics of included studies

First author, Year	Country	Study Design	No of patients	Type of surgery	Intervention^**a**^	Length of follow-up
a) Interventional studies						
Adamu, 2002 [13]	Ethiopia	RCT	153	BLTR and TTR	Surgical technique: BLTR vs TTR	3 months
Alemayehu, 2004 [14]	Ethiopia	RCT	982	BLTR	Personnel performing surgery: ophthalmologist vs IECWs	3 months and 6 months
Burton, 2005a [15]	The Gambia	RCT	451	PLTR	Perioperative azithromycin	6 months and 1 year
Burton, 2012 [16]	Ethiopia	RCT	1300	PLTR	Absorbable sutures vs silk sutures	1 year
Rajak, 2011a [17]						
Gower, 2011 [18]	Ethiopia	RCT	1452	BLTR	Perioperative azithromycin	6 weeks, 1 year and 3 years
West, 2006 [19]						
West, 2007 [20]						
Woreta, 2012 [21]						
Gower, 2013 [22]	Tanzania	RCT	1917	BLTR	TT clamp vs standard procedure	2 years
Rajak, 2011b [23]	Ethiopia	RCT	1300	PLTR	PLTR vs epilation	2 years and 4 years
Habtamu, 2015 [24]						
Habtamu, 2016 [25] 2017a [26] 2017b [27]	Ethiopia	RCT	1000	PLTR and BLTR	Surgical technique: PLTR vs BLTR	1 year
Habtamu, 2018 [28]	Ethiopia	RCT	1000	PLTR	Postoperative oral doxycycline vs placebo	1 year
b) Observational studies						
Ahmed, 2015 [29]	Egypt	Prospective non-comparative study	445	Anterior lamellar reposition	–	3 weeks, 3 months and 6 months
Assefa, 2008 [30]	Ethiopia	Prospective study	455	TTR	–	1 year
Bog, 1993 [31]	Tanzania	Prospective community-based study	94	Tarsal plate rotation	–	9–36 months
Bouazza, 2017 [32]	Ethiopia	Descriptive cross-sectional study	26	Anterior lamellar resection with lid margin splitting	–	6 months
Bowman, 2000 [33]	The Gambia	Retrospective Cross-sectional study	65	Tarsal rotation procedure	–	7 years
Burton, 2005b [34]	The Gambia	Retrospective cohort study	162	PLTR	–	3.5 years
Burton, 2010 [35]	The Gambia	Prospective cohort study	240	PLTR	–	4 years
el Toukhy, 2006 [36]	Egypt	Prospective study	493	BLTR	–	8 to 10 weeks
Kerie, 2010 [37]	Ethiopia	Cross-sectional survey	780	BLTR	–	3 months
Khafagy, 2012 [38]	Egypt	Prospective study	10	Combined eyelid splitting and selective cryotherapy	–	6 months
Merbs, 2005 [39] West, 2005 [40]	Tanzania	Retrospective cohort study	384	BLTR	–	> 18 months
Merbs, 2015 [41]	Tanzania	Preliminary data analysis from the PRET trial	145	BLTR	–	1 year
Ndoye, 1997 [42]	Senegal	Retrospective study	137	PLTR	–	2 years
Négrel, 2000 [43]	Morocco	Cross-sectional survey	740	BLTR	–	6 months
Pearson, 2013 [44]	Ethiopia	Cross-sectional survey	363	BLTR	–	11 months
Rajak,2010 [45]	The Gambia	Prospective study	356	PLTR	–	4 years
Rajak, 2013 [46]	Ethiopia	Prospective study	1300	PLTR	–	2 years
Woreta, 2009 [6]	Ethiopia	Prospective study within the STAR trial	448	BLTR	–	6 months

^a^Only for intervention studies

### Primary outcomes

#### Follow-up period of ≤3 months

In a study conducted in Ethiopia, 2.3% (59/2601) of operated eyelids were reported to have developed post-operative trichiasis by 6 weeks after BLTR [18].. An additional 1.3% (35/2601 eyelids) had eye closure defects, 1.2% (22/1881) had eyelid contour abnormalities and 10.5% (198/1881) had granulomata by 6 weeks [18]. In contrast, a previous study conducted in Egypt reported an incidence of 16.4% (98/599) amongst operated eyelids at 8 weeks after BLTR [36]. In the latter study, pre-operative corneal staining, corneal opacity and the use of silk sutures were identified as risk factors for early failure of BLTR. Other risk factors associated with post-operative trichiasis and other poor outcomes include surgeon’s experience and technique, short incision lengths, baseline severity of disease and epilation before surgery [18, 36].

Two intervention studies reported similar incidence of post-operative trichiasis; 10.4% [13] (12/115 patients) and 11.4% [14] (81/713 eyelids), while an observational study [37] conducted in Ethiopia reported a much higher incidence of post-operative trichiasis of 23.4% (308/1317 eyelids); 95% CI: 19.0–27.8), 6 months after BLTR. In the first study [13], the incidence of post-operative trichiasis was lower in the BLTR group compared to the TTR group (OR 0.84; 95% CI: 0.37–1.86), although this difference was not statistically significant. In the second intervention study [14], the incidence of post-operative trichiasis was higher in the intervention group (those operated on by an ophthalmologist) compared to those operated on by integrated eye care workers (OR 1.32; 95% CI: 0.83–2.11). In all 3 studies, a number of other poor outcomes were noted, including overcorrection, lid notching, pyogenic granuloma, local madarosis and conjunctival and eyelid inflammation [13, 14, 37].

#### Follow-up period: over 3 months but less than 1 year

**Fig. 2:**
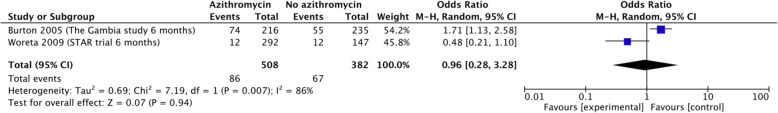
Summary of studies comparing peri-operative azithromycin to no azithromycin, with outcomes measured at 6 months

A randomized controlled trial conducted in The Gambia to investigate whether perioperative single-dose oral azithromycin could improve surgical outcomes after trichiasis surgery reported a cumulative incidence of 31.5% (129/410 patients) post-operative trichiasis 6 months after PLTR [15]. In this study, the incidence of post-operative trichiasis was higher in the intervention (azithromycin) group compared to the usual care (tetracycline eye ointment) group; 37.2% (74/199) versus 26.1% (55/211 patients) [15], but in multivariable logistic regression models there was no statistically significant difference between the groups. However, post-operative trichiasis was significantly associated with severity of preoperative disease and inter-surgeon variability [15]. In contrast, the Surgery for Trichiasis, Antibiotics to Prevent Recurrence (STAR) Trial conducted in Ethiopia reported a relatively low incidence of PTT; 4.6% (36/790 operated eyelids); 4.2% in the azithromycin group, 7.9% in the control group; OR 0.48; 95% CI: 0.21–1.10) 6 months after BLTR [6] (Fig. 2). In the latter study, surgeons had been highly selected and retrained before the intervention; while the Gambian study employed the available community ophthalmic nurses.

Other studies with 6 months follow-up produced estimates of the incidence of post-operative trichiasis of 6.2% (43/694 patients; 95% CI: 4.4–8) [14] and 15.8% (117/740 patients; CI not reported) [43] after BLTR. In a cross-sectional study conducted in Amhara Regional State of Ethiopia, 34 of the 363 patients (9.4%; 95% CI: 6.6–12.8) had post-operative trichiasis at 11 months after community-based trichiasis surgery with absorbable sutures. The prevalence of post-operative trichiasis reported in this study was lower than estimates reported in other observational studies although lid closure defects and lid notching were seen in 5.5% (20/363 patients) [95% CI: 3.4–8.4] and 16.8% (61/362 patients) [95% CI: 13.1–21.1] of cases respectively [44].

#### Follow-up period of 1 year

**Fig. 3:**
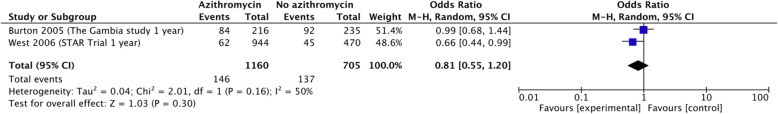
Summary of studies comparing peri-operative azithromycin to no azithromycin, with outcomes measured at 1 year

Among studies with a one-year follow-up period, the highest incidence of post-operative trichiasis at 41.3% (176/426 patients) was reported in the aforementioned Gambian study after PLTR surgery [15]. Unlike at 6 months follow-up, when the incidence of post-operative trichiasis was higher in the intervention (azithromycin) group than in the control group, at 1 year there were fewer cases of post-operative trichiasis in the intervention than in the control group; this difference was not statistically significant. In contrast, the STAR trial [19, 20] reported a relatively low incidence of post-operative trichiasis (7.6%; 107/1414 patients) 1 year after BLTR with significant difference seen between those who received peri-operative azithromycin and those in the control group (OR 0.66; 95% CI: 0.44–0.99). In combining the data from these two trials, those in the intervention (azithromycin) group were less likely to develop post-operative trichiasis (OR 0.81; 95% CI: 0.55–1.20) but the effect was not statistically significant (Fig. 3).

A number of other intervention studies reported estimates of > 10% for the incidence of post-operative trichiasis at 1 year after surgery: 17.4% (173/992 patients) [25–27], 19% (234/1236) [16, 17] and 12% (120/999) [28], as summarised in Table 2.

**Table 2:** Summary of intervention studies with outcomes measured at 1 year

Study	Intervention	Participants	OR; 95% CI
Habtamu 2016 [25]	PLTR vs BLTR	992	0.51 [0.36–0.72]
Rajak 2011 [16, 17]	PLTR with absorbable sutures vs silk sutures	1236	0.98 [0.73–1.30]
Habtamu 2018 [28]	PLTR with doxycycline vs placebo	999	0.93 [0.64–1.37]

#### Follow-up of more than one year

A retrospective cohort study conducted in Tanzania reported that 28% (176/630 eyes; CI not reported) of operated eyelids had developed post-operative trichiasis at 18 months after BLTR [39, 40]. At 2 years, several intervention studies reported relatively high incidence estimates of post-operative trichiasis following either BLTR or PLTR: 39.9% (1333/3343 eyelids) [22], 33.9% (412/1216 patients) [23] and 19% (235/1218 patients) [16, 17] as summarised in Table 3. One prospective cohort study [46] reported an incidence of 24.7% (315/1276 patients) 2 years after PLTR.

**Table 3:** Summary of intervention studies with outcomes measures at > 1 year

Study	Intervention	Participants	OR; 95% CI
Gower 2013 [22]	BLTR with TT clamp vs standard procedure	3343	1.32 [1.15–1.52]
Rajak 2011 [16, 17]	PLTR with absorbable sutures vs silk sutures	1218	0.99 [0.74–1.32]
Rajak 2011b [23]	PLTR versus epilation	1216	0.31 [0.24–0.42]

**Fig. 4:**
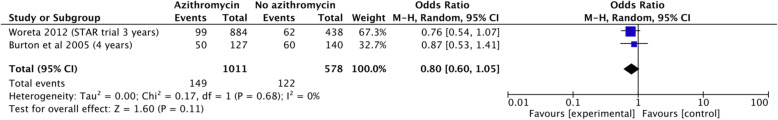
Summary of studies comparing peri-operative azithromycin to no azithromycin, with outcomes measured at 3 and 4 years

Similarly, at three and 4 years, high incidences of post-operative trichiasis (> 20%) were reported regardless of the surgical procedure used [24, 34, 35, 45]. In the Gambia study [15], the incidence of PTT was high (41%; 110/266 eyelids), 4 years after surgery. This was associated with increasing conjunctival inflammation at 4 years [23]. The STAR trial, however, was exceptional, reporting a cumulative incidence of post-operative trichiasis of only 12% (161/1322 patients) [19–21] in the 3 years after BLTR, with less post-operative trichiasis in the intervention group (OR 0.76; 95% CI: 0.54–1.07] – Fig. 4

#### Comparison between surgical procedures and study types

**Fig. 5:**
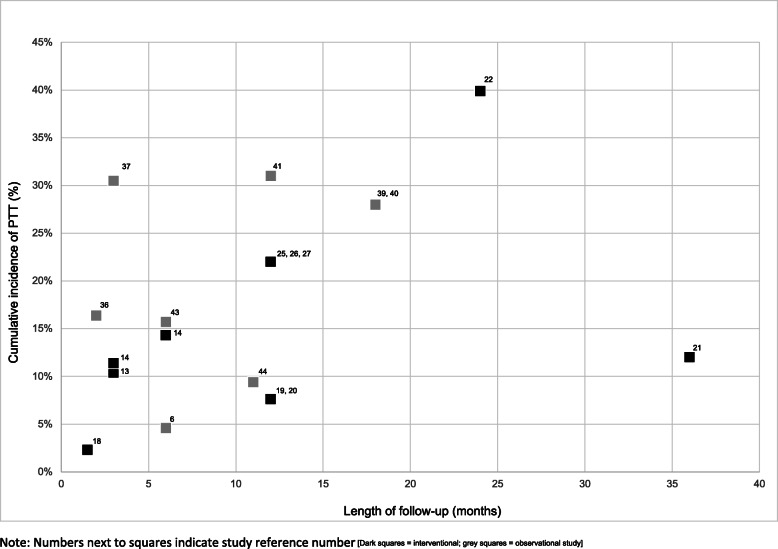
Incidence of PTT among patients who had BLTR, by type of study and duration of follow-up

**Fig. 6:**
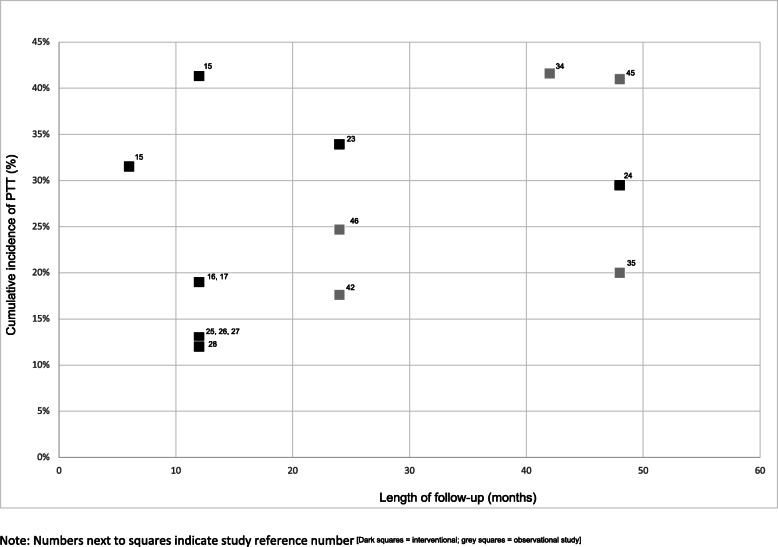
Incidence of PTT among patients who had PLTR, by type of study and duration of follow-up

The incidence of post-operative trichiasis was high (> 20%) across different follow-up periods regardless of the surgical procedure used (Figs. 5 and 6). Only one study provided a direct comparison between the two most common procedures for trichiasis surgery – PLTR and BLTR [25]. In this study conducted in Ethiopia, PLTR was found to yield a lower incidence of post-operative trichiasis compared to BLTR (OR 0.51; 95% CI: 0.36–0.72).

In terms of type of study, the incidence of post-operative trichiasis varied in both interventional and observational studies. Generally, however, the cumulative incidence of PTT was lower in interventional studies [7, 17, 22, 24, 25, 27] than in observational studies [29, 30, 33–35, 37, 39–41, 45, 46] (Figs. 5 and 6). Amongst ten interventional studies reporting outcomes ≤6 months after surgery, the four that either re-trained or specifically selected surgeons had the lowest post-operative trichiasis incidence estimates.

At 3–6 months follow-up, two interventional studies [13, 48] reported comparable incidences of post-operative trichiasis: 11.4% (81/713 patients) and 14.3% (124/901 patients) at 3 and 6 months respectively following BLTR; 10.4% (12/115 eyelids) 3 months after BLTR and 12.3% (15/122 eyelids) 3 months after transverse tarsotomy and lid margin rotation group [13]). This was significantly lower than the incidence reported in two observational studies over the same length of time: 44.7% (336/752 eyelids) [29] and 30.5% (238/780 patients) [37].

## Discussion

Our systematic review suggests that there is a huge disparity between policy and practice with respect to the incidence of post-operative trichiasis in Africa. WHO recommends that national trachoma programmes strive to achieve ≤10% post-operative trichiasis by 1 year after surgery [8], but in the eight studies we identified that had one-year data, only one had an incidence of post-operative trichiasis ≤ 10% [19]. This suggests that there is a need to put in place or strengthen existing measures to improve the proportion of patients achieving satisfactory outcomes. A logical approach would be for national trachoma programmes to establish and implement policies and systems to follow up patients, assess surgical outcomes and maximize the performance of individual surgeons through post-surgical audits with provision of additional support, where needed.

BLTR and PLTR are currently the most commonly used procedures, with evidence presented elsewhere suggesting that they produce better outcomes than other techniques [13, 23, 24]. Only one recent study [25] has directly compared the two. Additional research is warranted to confirm the apparent superiority of PLTR [49].

Our study has clear weaknesses for estimating the rate and determinants of success in trichiasis surgery programs; these should be acknowledged before further conclusions are drawn. We did not collect program-level data, but rather collated reported post-operative trichiasis incidence estimates from the literature, with referenced papers derived from the experience of academically supported settings over three decades. The contemporary incidence of bad outcomes when surgery was being routinely performed in unselected program delivery environments could easily have been higher (or lower) than noted, and there are too few data points and too many potential explanatory variables to try to infer possible changes in incidence over time.

Data on the phenotype of post-operative trichiasis were generally not published; the potential implications for vision of having one or two peripheral trichiatic eyelashes are likely to be considerably less than those of large numbers of central trichiatic eyelashes. It is also important to note that most studies included in this review were conducted before publication of the International Coalition for Trachoma Control’s training and supervision guidelines [50, 51], and before mannequin-based surgical simulation training [52] became available. These measures are likely (though formally unproven) to improve the outcomes of trichiasis surgery.

How else could success rates be optimized? A large randomized controlled trial in Ethiopia suggested that a single dose of peri-operative azithromycin was associated with a 33% reduction in post-operative trichiasis by 3 years after surgery, compared with topical tetracycline prescribed for 6 weeks [19–21]. These data differ from a smaller clinical trial comparing peri-operative oral azithromycin with two post-surgical weeks of topical tetracycline conducted in The Gambia [15], where no significant difference in outcomes was observed. One important difference between those two trials was the absence of trial-specific training and standardization of surgeons in the Gambian study, which might have resulted in a higher incidence of post-operative trichiasis; significant variability between surgeons was noted, with incidence of post-operative trichiasis ranging by surgeon from 0 to 83%. The Ethiopian study [19] used surgeons who had been trained and certified by the study team prior to the start of the trial. One interpretation of this combination of findings would be that, though azithromycin might have a protective effect against post-operative trichiasis, it cannot overcome shortfalls in training, standardization and supervision of surgeons. Other differences between the two environments might also, or alternatively, be responsible. The mechanism for azithromycin having an impact, if it does, is not necessarily clear: it could potentially relate to either the anti-inflammatory or anti-infective action of the macrolide.

In another randomized controlled trial conducted in Ethiopia [17], there was no evidence that use of absorbable sutures was associated with a lower incidence of post-operative trichiasis at 1 year after surgery compared to the use of silk (although absorbable sutures were found to reduce the incidence of granulomata) [16, 17]. Despite careful standardisation, there was significant variability in outcomes between different surgeons in this trial. To protect patients, operator-dependent factors, including dexterity, handedness, visual acuity, rigor of training and certification, supervision and motivation, must always be considered possible contributors to the occurrence of post-operative trichiasis. The tendency noted in the current review for post-operative trichiasis incidence to be lower in interventional than in observational studies was most marked for interventional studies where surgeons were re-trained prior to initiation of the study, reinforcing the (perhaps expected) point that greater skill levels drive better results.

## Conclusion

Since 1990, published estimates suggest that anatomical outcomes of the surgical management of trachomatous trichiasis are worse than expected by WHO. More effort needs to be made to better understand the routine incidence, phenotype and determinants of post-operative trichiasis. This knowledge is critical to help design strategies to minimize its occurrence. In the meantime, stringent adherence to recommended follow-up schedules [53] is recommended to ensure that patients experiencing sub-optimal outcomes are detected early and offered appropriate care.

## Supplementary information

**Additional file 1.**

**Additional file 2.**

## Data Availability

The datasets used and/or analysed during the current study available from the corresponding author on reasonable request.

## Abbreviations

BLTR: Bilamellar tarsal rotation
CI: Confidence interval
CINAHL: Cumulative Index to Nursing and Allied Health Literature
IECWs: Integrated eye care workers
OR: Odds ratio
PLTR: Posterior lamellar tarsal rotation
PTT: Post-operative trichiasis
SAFE: Surgery, Antibiotics, Facial cleanliness, Environmental improvement
STAR: Surgery for Trichiasis, Antibiotics to Prevent Recurrence
TTR: Transverse tarsotomy with lid margin rotation
WHO: World Health Organization
