# Supplementary material for: Systematic review of the incidence of post-operative trichiasis in Africa
Source: BMC Ophthalmol. 2020 Nov 17;20:451. doi: 10.1186/s12886-020-01564-0 (PMC7670604; doi:10.1186/s12886-020-01564-0)
Supplement: Supplementary file 1 — Additional file 1. [file 12886_2020_1564_MOESM1_ESM.pdf]

Search Strategy: PubMed

((("trichiasis"[MeSH Terms] OR "trichiasis"[All Fields]) OR "trichiasis"[MeSH Terms]) OR ("entropion"[MeSH Terms] OR "entropion"[All Fields])) OR "entropion"[MeSH Terms]
