# Supplementary material for: Systematic review of the incidence of post-operative trichiasis in Africa
Source: BMC Ophthalmol. 2020 Nov 17;20:451. doi: 10.1186/s12886-020-01564-0 (PMC7670604; doi:10.1186/s12886-020-01564-0)
Supplement: Supplementary file 2 — Additional file 2. [file 12886_2020_1564_MOESM2_ESM.docx]

**Supplementary Materials: List of excluded studies**

| **Author(s)** | **Year** | **Reason for exclusion** |
| --- | --- | --- |
| **Conducted outside Africa** | |  |
| Reacher *et al*. | 1990 | Conducted in Oman |
| Cruz *et al*. | 1991 | Conducted in Brazil |
| Reacher *et al*. | 1992 | Conducted in Oman |
| Stades *et al*. | 1993 | Conducted in Netherlands |
| Yeung *et al*. | 1997 | Conducted in Hong Kong |
| Beigi b | 2001 | Conducted in UK |
| Bujger *et al*. | 2004 | Conducted in Croatia |
| Dhaliwal *et al*. | 2004 | Conducted in India |
| Zhang *et al*. | 2004 | Conducted in Nepal |
| Sadiq & pai | 2005 | Conducted in Oman |
| Khandekar *et al*. | 2006 | Conducted in Oman |
| Moosavi *et al*. | 2007 | Country unspecified |
| Monga *et al*. | 2008 | Conducted in India |
| Bleyen & dolman | 2009 | Conducted in Canada |
| Sakarya *et al*. | 2010 | Conducted in Turkey |
| Ali *et al*. | 2012 | Conducted in Pakistan |
| Nakauchi *et al*. | 2012 | Conducted in Japan |
| Yagci & palama | 2012 | Conducted in Turkey |
| Barr *et al*. | 2014 | Conducted in Australia |
| Cruz *et al*. | 2015 | Conducted in Saudi Arabia |
| Ferraz *et al*. | 2017 | Conducted in Brazil |
| Russell & seiff | 2017 | Conducted in USA |
| Sendul *et al*. | 2018 | Conducted in Turkey |
| **Different outcome** |  |  |
| Schémann *et al*. | 1997 | Prevalence of trachoma in the region |
| Bowman *et al*. | 2001 | Incidence of trichiasis |
| Ezz *et al*. | 2001 | Prevalence of trachoma |
| Frick *et al*. | 2001 | Cost of illness of untreated trichiasis |
| Bowman *et al*. | 2002 | Progression of trichiasis and corneal scarring |
| Wolle *et al*. | 2010 | Physical functioning of trichiasis surgery patients |
| Rajak *et al*. | 2011 | Corneal opacity |
| Gower *et al*. | 2012 | New grading scheme for eyelid contour abnormality |
| Merbs *et al*. | 2012 | Description of the new of TT clamp for trichiasis surgery |
| Merbs *et al*. | 2012 | Immediate post-operative eyelid contour |
| Li *et al*. | 2013 | Anti-scarring properties of doxycycline |
| Schein *et al*. | 2016 | Corneal sensitivity |
| Bickley *et al*. | 2017 | Uptake of surgery |
| Habtamu *et al*. | 2015 | Economic poverty |
| Habtamu *et al*. | 2015 | Vision and health related quality of life |
| Mousa *et al*. | 2015 | Uptake of trichiasis surgery |
| Oktavec *et al*. | 2015 | Perceptions of surgery 24 months |
| Smith *et al*. | 2015 | Prevalence of TT and CO |
| Thompson *et al*. | 2015 | Health beliefs and perceptions |
| Burr *et al*. | 2016 | Prevalence of TF and TT |
| Habtamu *et al*. | 2016 | Impact of trichiasis surgery on vision |
| **Reviews & editorial articles** | |  |
| Reacher & taylor | 1990 | Review |
| Elder & collin | 1997 | Editorial article |
| Bowman rj | 1999 | Editorial article |
| Ruban & baggio | 2003 | Article |
| Burton & solomon | 2004 | Article |
| Yorston *et al*. | 2006 | Systematic review |
| Lin & lietman | 2007 | Editorial |
| Kirkwood *et al*. | 2011 | Article |
| Rajak *et al*. | 2012 | Major review |
| Lockwood & stern | 2014 | Systematic review |
| Burton *et al*. | 2015 | Systematic review |
| Habtamu & burton | 2015 | Article |
| Ramadhani *et al*. | 2016 | Review |
| Solomon aw, 2016 | 2016 | Comment |
| **Not trachomatous trichiasis** | |  |
| Scheepers *et al*. | 2010 | Involutional lower eyelid entropion |
| Cevallos *et al*. | 2012 | Conjunctival bacterial infection |
| Burn *et al*. | 2017 | Podoconiosis |
| **Other reasons** |  |  |
| Zbiba *et al* | 1993 | Internal inconsistencies |
